# Proliferative diabetic retinopathy and diabetic macular edema are two factors that increase macrophage-like cell density characterized by en face optical coherence tomography

**DOI:** 10.1186/s12886-023-02794-8

**Published:** 2023-02-01

**Authors:** Wenyu Wang, Gongpeng Sun, Amin Xu, Changzheng Chen

**Affiliations:** Department of Ophthalmology, Renmin Hospital of Wuhan University, Wuhan, China

**Keywords:** En face OCT, Retina, Diabetic retinopathy, Macrophage-like cells

## Abstract

**Background:**

Macrophage-like cells (MLCs) located at the ILM were observed in live human retinas using adaptive optics optical coherence tomography (OCT) as well as clinically-used OCT. The study aimed to quantitatively analyzing MLCs at the vitreoretinal interface (VRI) in diabetic retinopathy (DR) using en face OCT and swept-source optical coherence tomography angiography (SS-OCTA).

**Methods:**

190 DR eyes were included in the study, with 70 proliferative diabetic retinopathy (PDR) eyes and 120 non- proliferative diabetic retinopathy (NPDR) eyes. Sixty-three eyes from normal subjects were included as controls. MLCs were visualized in a 5 μm en face OCT slab above the VRI centered on the fovea. Mann-Whitney U test and Kruskal-Wallis H test were used to compare the OCTA parameters and the MLC parameters among groups. We evaluated the MLC density among groups on binarized images after image processing. We also investigated the relationship between MLC density and other OCT parameters including retina thickness and vessel density (VD).

**Results:**

The MLC density significantly increased in PDR eyes (PDR vs. NPDR, 8.97 (8.40) cells/mm^2^ vs.6.14 (8.78) cells/mm^2^, *P* = 0.013; PDR vs. normal, 8.97 (8.40) cells/mm^2^vs. 6.48 (6.71) cells/mm^2^, *P* = 0.027) and diabetic macular edema (DME) eyes (DME vs. without DME, 8.94 (8.26) vs.6.09 (9.00), *P* = 0.005). After adjusting for age and gender, MLC density in NPDR eyes negatively correlated to VD of deep capillary plexus (DCP) (*P* = 0.01).

**Conclusions:**

SS-OCTA is a non-invasive and simple method for the characterization of MLCs at the VRI. PDR and DME are two factors that increase MLC density. MLC density also correlated with VD.

**Supplementary Information:**

The online version contains supplementary material available at 10.1186/s12886-023-02794-8.

## Background

Diabetic retinopathy (DR) is a leading cause of vision loss and lead to heavy economic burden in many countries [1]. 40% of people with type 2 diabetes and nearly 86% with type 1 diabetes have DR. Hyperglycemia and diabetes duration are two of the risk factors for DR [2, 3]. Structural and functional changes in the retinal vasculature related to DR. The development of macula edema is considered as a direct consequence of inner blood–retinal barrier (iBRB) breakdown [4]. In addition, some evidence has demonstrated that inflammation and immune system dysregulation accompanied the progression of DR [5, 6]. Besides the increased level of inflammatory factors, previous study illustrated that microglia were activated due to prolonged hyperglycemia and tissue stress [7]. Study in the mouse model of oxygen-induced retinopathy found that microglia occupy ischemic and neovascular regions of the retina and play roles in the development of neovasculature [8]. Clinical optical coherence tomography (OCT) also found that retinal hyperreflective spots increased and reached the outer retinal layer with DR progression. These hyperreflective spots were considered as activated microglia in DR [9]. The activation and migration of microglia were also found in paraffin sections from DR patients’ retina [10]. These findings suggested that reactive microglia participated in DR and promoted the progression of DR to the proliferative stage [7].

Macrophages in the vitreous humor were known as hyalocytes, parts of which located above internal limiting membranes (ILM) [11–13]. In previous studies, both microglia and hyalocytes were considered as originating from the monocyte-macrophage system and played an important part in several retinal diseases such as glaucoma [14], DR [5], uveitis [7], idiopathic epiretinal membrane [15] and age-related macular degeneration [16]. Recently, some studies have reported that macrophages located at the ILM were observed in live human retinas using adaptive optics OCT as well as clinically-used OCT [17–25]. These studies also described the distribution and dynamics of the cells [17]. Furthermore, other studies with a small sample size observed an increase in macrophage-like cells (MLCs) at the vitreoretinal interface (VRI) in pathological processes of retina [25, 26].

Based on studies using animal models and a little available research on human retina, we presumed that macrophages involved in the pathological process of DR. The current study collected patients of non-proliferative diabetic retinopathy (NPDR) and proliferative diabetic retinopathy (PDR), aiming to observe changes in the quantity, distribution characteristics, and morphology of MLCs through clinical swept source OCT (SS- OCT) and en face OCT.

## Methods

### Subjects

This retrospective cross-sectional study included PDR and NPDR patients and normal subjects between April 2021 to May 2022 at the Eye Center of the Renmin Hospital of Wuhan University in Wuhan, China. The study was approved by the Institutional Review Board of the Renmin Hospital of Wuhan University (WDRY2021-k162) and conducted in accordance with the tenets of the Declaration of Helsinki. Informed consent was obtained from all participants. Both eyes of all participants underwent comprehensive ophthalmologic examinations, including visual acuity, fundus fluorescein angiography (FFA), color fundus photography (CFP) and slit lamp examination. Diabetic maculae edema (DME) was diagnosed by OCT volumes centered on macular. Some apparent retinal thickening or hard exudates in posterior pole was considered as DME. Diagnosis and staging of DR was based on FFA, CFP or ultra-wide field images. Eyes with neovascularization and/or vitreous/preretinal hemorrhage were considered as PDR eyes. Eyes with microaneurysms, intraretinal hemorrhages, venous beading, intraretinal microvascular abnormalities and no signs of proliferative retinopathy were NPDR eyes. All diagnoses were performed by an experienced ophthalmologist. Exclusion criteria included: (1) prior surgical procedures such as pars plana vitrectomy; (2) concomitant with other retinal diseases (glaucoma, non-arteritic anterior ischemic optic neuropathy, uveitis, pathologic myopia, etc.); (3) vitreous hemorrhage, cataract, or poor fixation affecting imaging quality; (4) eyes with large area of epiretinal membrane (ERM) affecting image analysis; (5) subjects with refractive error > 3.0 diopters(D) or < − 6.0 D; (6) eyes received intravitreal injection of steroids or received anti-VEGF therapy within the last 3 months.

### OCTA image acquisition

OCTA images were acquired using a commercial SS-OCTA instrument (VG100, SVision Imaging, Ltd., Luoyang, China). OCTA was performed using a raster scan protocol of 512 (horizontal) × 512 (vertical) which covered an area of 6 mm × 6 mm centered on the fovea. OCT B-scans corresponding to OCTA images were acquired at the same time. OCTA images of the peripheral retina were taken in the same scan mode as the macular area by guiding eye position. Only the OCTA images centering on the fovea were applied to quantitative analysis of MLC density. Repeated images (range, 3–5 repeats) at the same location were acquired to increase the credibility of the results. The MLC layer was segmented by a 5-μm OCT slab located from 5 to 10 μm above the ILM to display the MLCs clearly. The segmentation lines were adjusted manually if needed.

### Retinal parameter acquisition

VD of the deep capillary plexus (DCP), superficial capillary plexus (SCP), mean retina thickness (MRT), and central fovea thickness (CFT) were also automatically measured in 6 mm × 6 mm OCTA images centered on the fovea by the built-in software of the SS-OCTA instrument (version 1.36.4). OCTA parameters including VD of DCP, VD of SCP, MRT and CFT were further separately measured in the parafoveal region (1–3 mm), and perifoveal region (3–6 mm).

### Image processing

The en face OCT images were processed by ImageJ software. First, we used the White Top Hat algorithm, the Subtract Background and Gaussian Blur to remove some artifacts. Second, the Trainable WeKa Segmentation was used to mark MLCs. Finally, we applied the Analyze Particles tool to analyze the number of MLCs A semi-automated macro program for image processing were used. Specific processes were shown in the supplement (S1).

### Statistical analysis

We used SPSS Statistics (version: 26.0; IBM Corp., Armonk, NY, USA). Continuous variables coincided with normal distribution are presented as the mean ± standard deviation (SD). Continuous variables not coincided with normal distribution are presented as median (interquartile range [IQR]). After using the Shapiro-Wilk test to test the normality of the data and Levene’s test for the homogeneity test of variance, we performed independent samples Mann-Whitney U test and Kruskal-Wallis H test to compare the OCTA parameters and the MLC parameters among PDR eyes, NPDR eyes and the control group. Chi-square test or Fisher’s exact test were used for categorical variables. Based on the data distribution type, the Spearman correlation coefficient and multiple linear regression were used to verify the correlation between the MLC density and the OCTA parameters in NPDR and PDR groups. Generalized estimating equations (GEE) were used to eliminate the effect of repeatability and relevance in data.

## Result

190 DR eyes from 128 patients were included in the study (mean age, 56.47 ± 10.41, 47 females),including bilateral eyes from 62 patients and unilateral eyes from 66 patients. Among the included eyes, PDR and NPDR eyes accounted for 36.8% (70/190) and 63.2% (120/190) respectively. There were 77 eyes with DME (40.5%) and 53 eyes received panretinal photocoagulation (PRP) (27.9%). We also included 63 normal eyes from 63 normal subjects as the control group (mean age, 55.56 ± 12.12, 25 females).

**Fig. 1:**
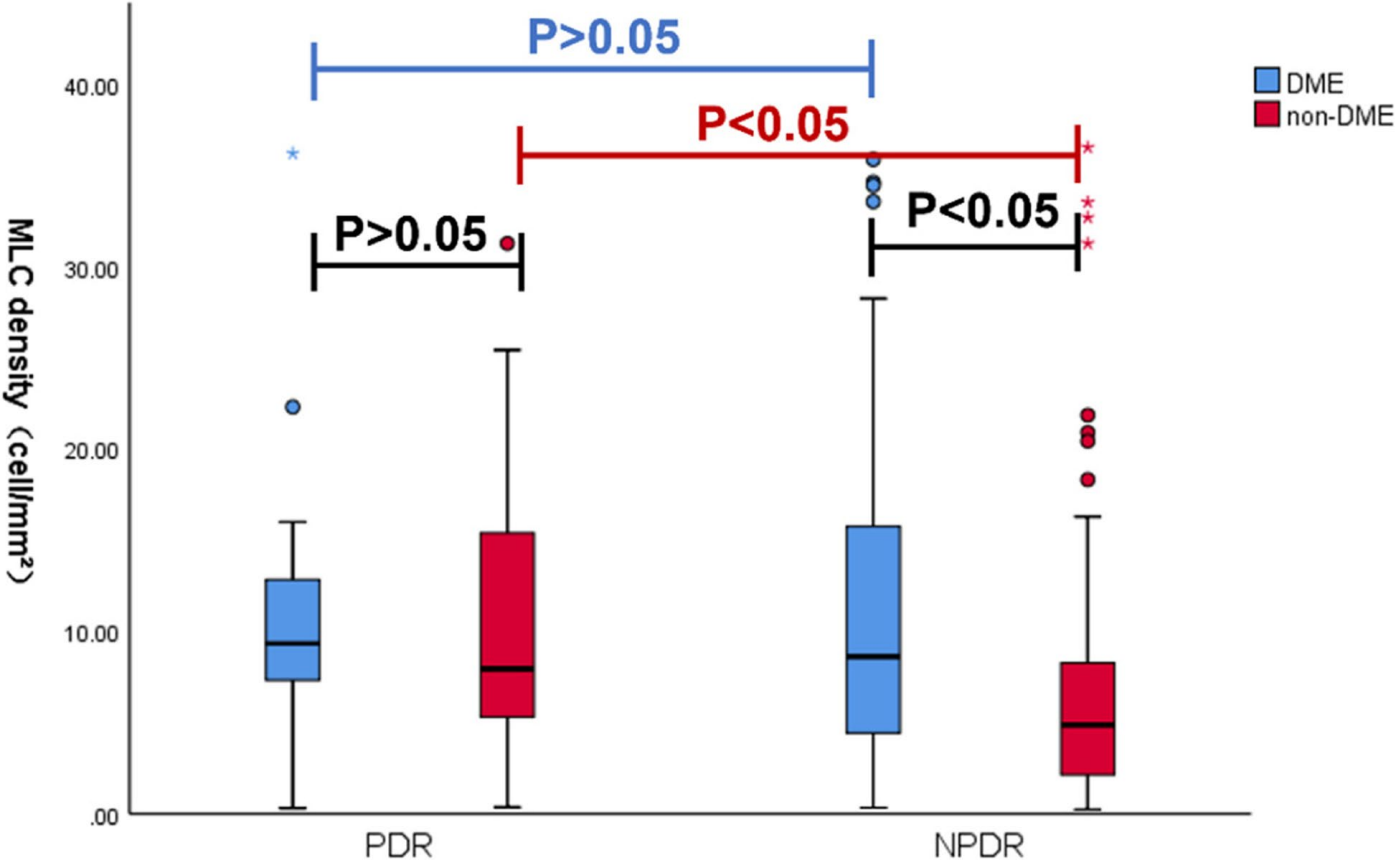
Comparison of MLC density between groups of different DR stages (PDR vs. NPDR) and groups with or without DME. DME only increased the number of MLCs in NPDR group, not in the PDR group. In eyes without DME, proliferation stage increased number of MLCs. In eyes with DME, no similar effect of proliferation stage was found

The MLC density and OCTA parameters are summarized in Table 1. There was no significant difference in MLC density between NPDR eyes and controls (6.14 (8.78) cells/mm^2^ vs. 6.48 (6.71) cells/mm^2^, *P* = 0.566). Compared with NPDR eyes and normal eyes, MLC density in PDR group increased significantly (8.97 (8.40) cells/mm^2^ vs.6.14 (8.78) cells/mm^2^, *P* = 0.013; 8.97 (8.40) cells/mm^2^vs. 6.48 (6.71) cells/mm^2^, *P* = 0.027). Examples are shown in Fig. 3A, E, B, F, G&H. The presence of DME also affected MLC density. DR eyes with DME showed more MLCs than those without DME (8.94 (8.26) vs.6.09 (9.00), *P* = 0.005, Fig. 3B&F vs. C&G). The results were shown in Fig. 1 after taking disease stages into account. The effect of DME on MLC density was only present in the NPDR group (*P* = 0.002). In PDR eyes, no significant difference in MLC density was found between eyes with DME and without DME (*P* = 0.713). And MLC density was comparable between NPDR with DME (*n* = 50) and PDR (*n* = 70) (8.60 (11.07) vs 8.97 (8.40), *P* = 0.91). Considering that we included bilateral eyes giving rise to non-independent, correlated data points, GEE was applied to eliminate the effect of correlation. PDR (OR = 2.47, *P* = 0.040) and DME (OR = 5.73, *P* = 0.026) are two factors for the increase in MLC density after adjusting for correlation effect. Also, there was no statistical difference in MLC density comparing with eyes received PRP with those not received PRP in either NPDR group (7.49 (7.01) vs. 5.85 (9.06), *P* = 0.052) or PDR group (11.52 (13.40) vs. 8.79 (7.15), *P* = 0.95). Statistical analysis of the DR eyes and normal eyes in Mann-Whitney U test found that the MRT and CFT were significantly thicker in DR eyes (*P* < 0.001) and the VD of DCP decreased in DR eyes (*P* < 0.001). Also, VD of DCP also decreased with disease progression (NPDR vs. PDR, 47.88 (8.05) vs. 43.68 (11.62), *P* < 0.001). And there is no significant difference between PDR and NPDR groups in other OCTA parameters.

**Table 1:** OCTA parameters and MLC density in control and DR groups

	Control	DR			
		NPDR	PDR	*P*^††^	P^*^
DME, n (%)	–	50 (41.7)	27 (38.57)	0.675^#^	–
PRP, n (%)	–	31 (25.8)	22 (31.42)	0.407^#^	–
MLC density (cells/mm^2^), median (IQR)	6.48 (6.71)	6.14 (8.78)	8.97 (8.40)	**0.013**^†^	**0.025**
VD of DCP (%), median (IQR)	55.88 (3.95)	47.88 (8.05)	43.68 (11.62)	**< 0.001**^†^	**< 0.001**
VD of SCP (%), mean ± SD	50.07 ± 5.01	51.00 ± 4.82	51.42 ± 6.91	0.455^†^	0.35
MRT (μm), median (IQR)	291.49 (16.38)	316.79 (52.83)	319.52 (53.85)	0.431^†^	**< 0.001**
CFT (μm), median (IQR)	205.28 (18.54)	221.08 (115.19)	215,93 (54.90)	0.432^†^	**< 0.001**

*DME* diabetic macular edema, *PPR* panretinal photocoagulation, *MLC* macrophage-like cell, *VD* vessel density, *DCP* deep capillary plexus, *SCP* superficial capillary plexus, *MRT* mean retina thickness, *CFT* central fovea thickness, *SD* standard deviation, *IQR* interquartile range

^††^comparison between NPDR and PDR

^†^Mann-Whitney U test

^#^Chi-square test

^*^comparison among control group, NPDR and PDR eyes using nonparametric Kruskal–Wallis H test

**Fig. 2:**
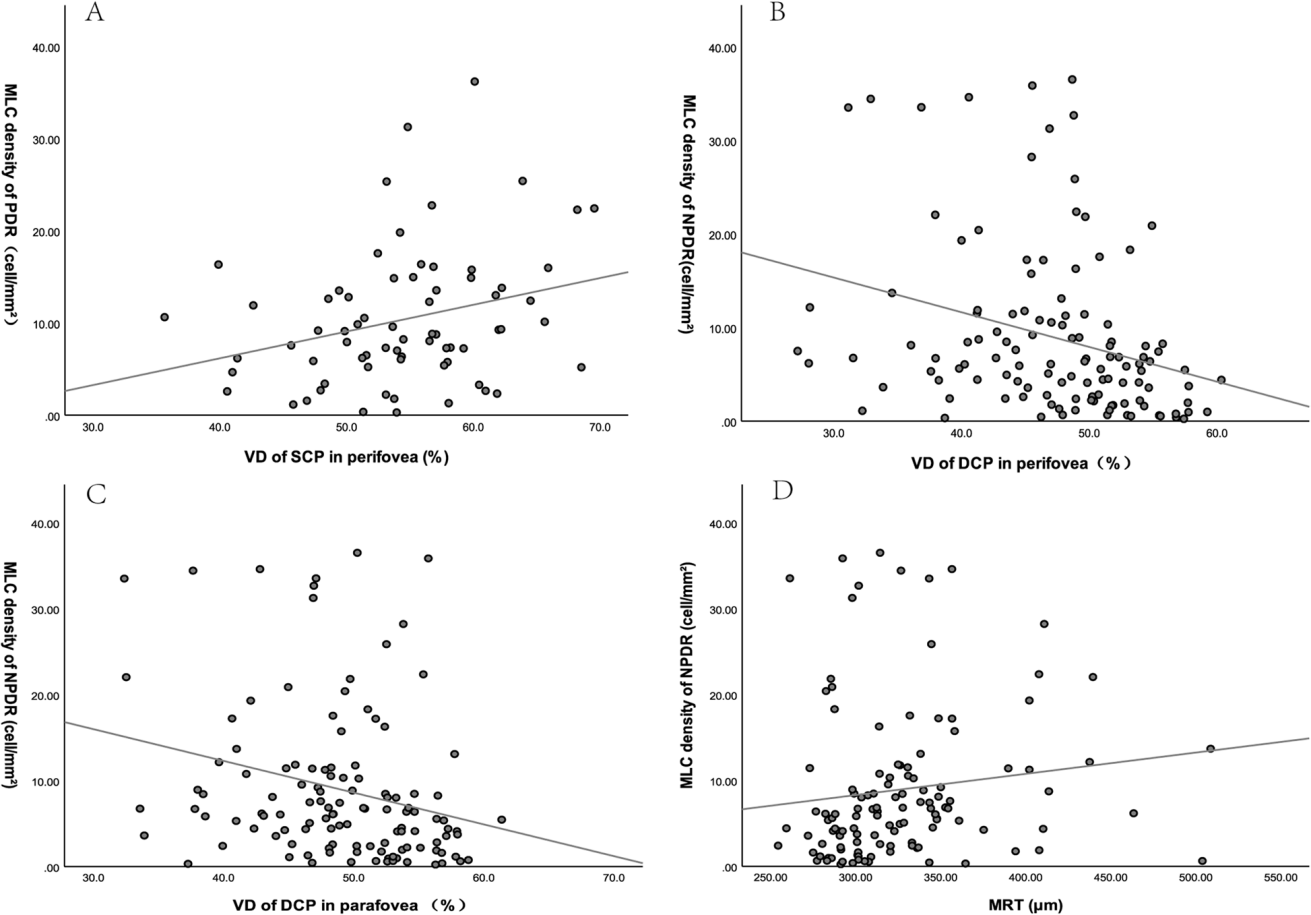
**A** The positive correlation between MLC density and VD of SCP in perifovea in PDR group. **B** & **C** The negative correlation between MLC density and VD of DCP in perifovea and parafovea in NPDR group. **D** The positive correlation between MLC density and MRT in NPDR group

We also investigated that whether MLC density associated with VD and retina thickness. The results seemed to be different in PDR and NPDR groups, which was summarized in Tables 2 and 3. In PDR group, MLC density of PDR only correlated with VD of SCP in perifovea after adjusting for age and sex (*r* = 0.292, *P* = 0.016). In NPDR eyes, MLC density positively correlated to MRT (*r* = 0.27, *P* = 0.003) and negatively correlated to VD of DCP (*r* = − 0.388, *P* < 0.001). After adjusting for age and gender, only VD of DCP still negatively correlated with MLC density in NPDR (*P* = 0.01). The results of the correlation analysis are shown in the Fig. 2.

**Table 2:** Correlation between MLC density and OCTA parameters in PDR eyes

		r	*P*	r^*^	P^*^
CFT		0.007	0.995	− 0.008	0.951
MRT		0.119	0.328	0.012	0.926
VD of DCP	parafovea	−0.103	0.395	−0.06	0.624
	perifovea	−0.14 + 9	0.219	−0.13	0.29
VD of SCP	parafovea	0.108	0.375	0.138	0.262
	perifovea	0.268	**0.025**	0.292	**0.016**

*CFT* central fovea thickness, *MRT* mean retina thickness, *VD* vessel density, *DCP* deep capillary plexus, *SCP* superficial capillary plexus

^*^after controlling for age and gender

**Table 3:** Correlation between MLC density and OCTA parameters in NPDR eyes

		r	*P*	r^*^	P^*^
CFT		0.08	0.385	0.041	0.662
MRT		0.27	**0.003**	0.139	0.133
VD of DCP	parafovea	−0.315	**<0.001**	−0.248	**0.007**
	perifovea	−0.375	**<0.001**	−0.262	**0.004**
VD of SCP	parafovea	−0.172	0.061	−0.040	0.668
	perifovea	0.095	0.301	0.133	0.152

*CFT* central fovea thickness, *MRT* mean retina thickness, *VD* vessel density, *DCP* deep capillary plexus, *SCP* superficial capillary plexus

^*^after controlling for age and gender

**Fig. 3:**
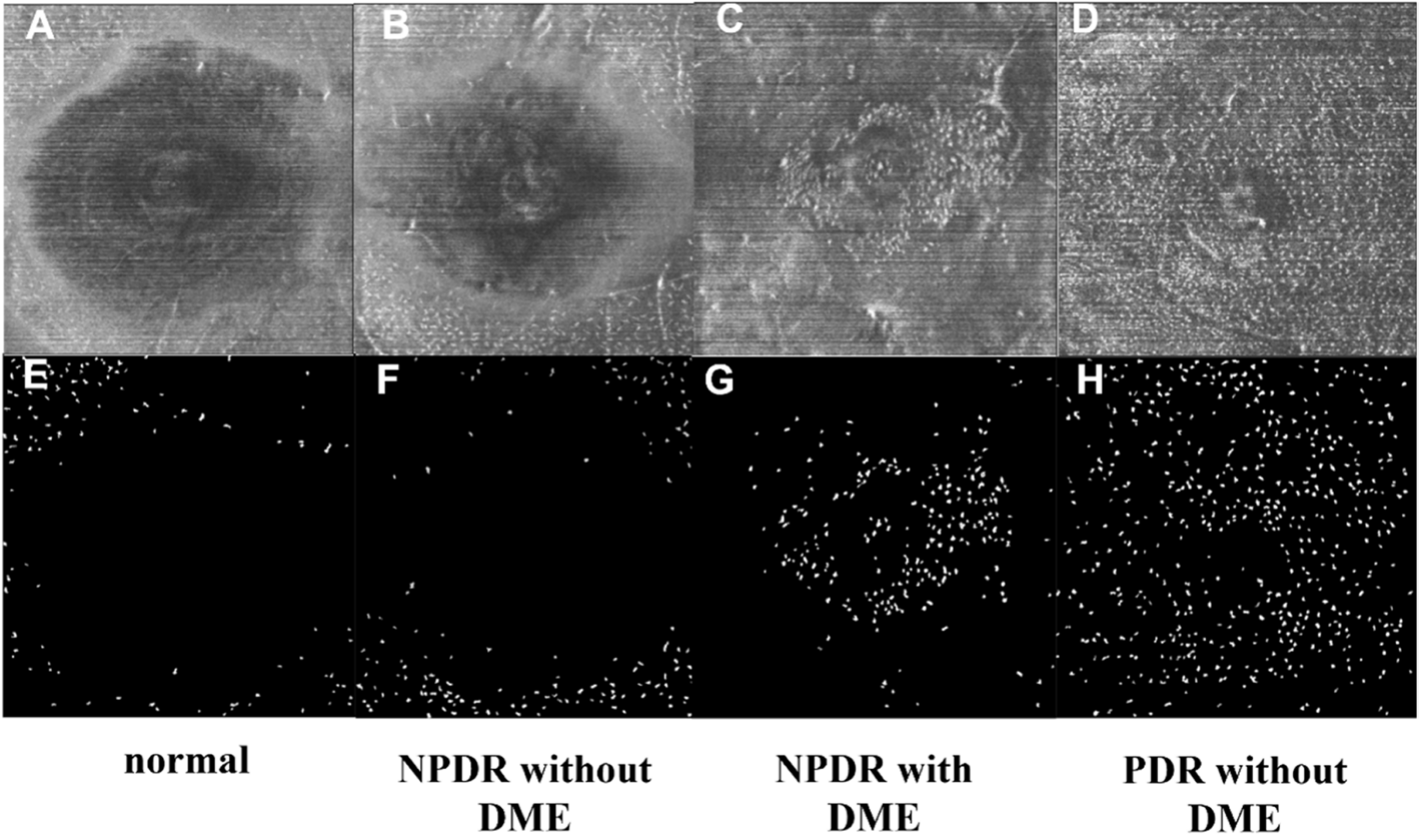
En face OCT slabs of MLCs and binarized images from control and DR eyes. **A** & **E** en face OCT image and binarized image from a 52-year-old female subject with no retina disease. **B** & **F** en face OCT image and binarized image from 33-year-old male NPDR patients without DME. **C** & **G** en face OCT image and binarized image from 65-year-old female NPDR patient with DME. More MLCs were found in parafoveal area. **D** & **H** en face OCT image and binarized image from a 70-year-old female patient with PDR. Her contralateral eye underwent pars plana vitrectomy for vitreous hemorrhage. MLC density increased in the PDR eyes

**Fig. 4:**
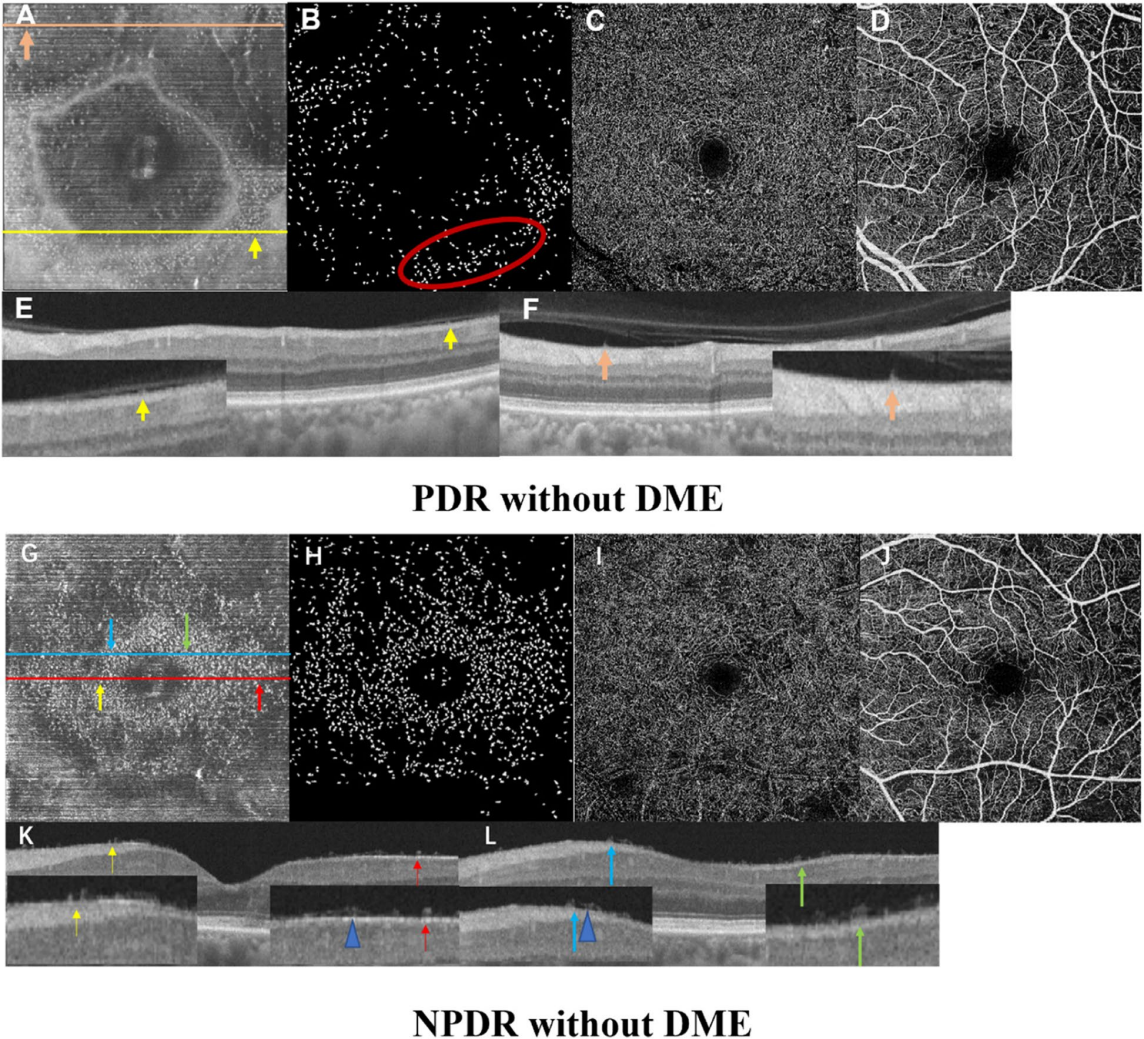
Images from PDR and NPDR patients **A-F** images from a 60-year-old man with PDR without DME. **A** & **B** En face OCT slab of MLCs and binarized image. The distribution of MLCs was uneven. Some of them gathered around retinal vessels (red ellipse). **C** & **D** OCTA images of DCP and SCP. Microaneurysms and small pieces of nonperfusion area are visible. **E** & **F** B-scans and magnified images of yellow and pink lines in **A**. The arrows in **E** & **F** correspond to arrows of the same color in **A**. MLCs appeared as small protrusions located at the VRI on B scans. **G-L** images from a 63-year-old man with NPDR without DME. **G** & **H** En face OCT slab of MLCs and binarized image. MLCs gathered in fovea area. **I** & **J** OCTA images of DCP and SCP. **K** & **L** B-scans and magnified images of red and blue lines in **G**. The arrows in **K** & **L** correspond to arrows of the same color in **G**. MLCs appeared as small protrusions located at the VRI on B scans (arrows). Connections appear among some adjacent MLCs (blue triangle)

**Fig. 5:**
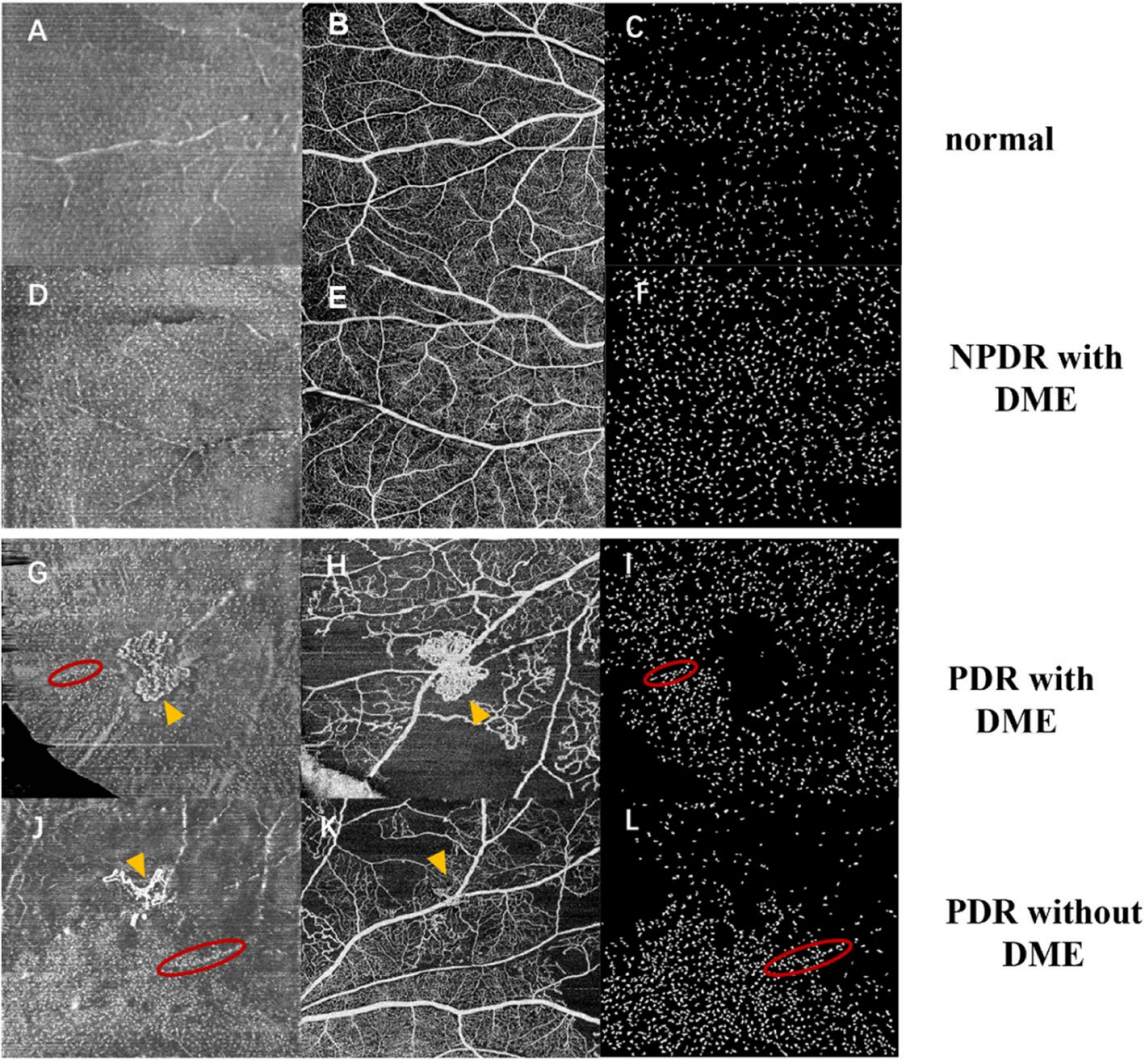
En face OCT slabs of MLCs, OCTA and binarized images of MLCs in peripheral retina. **A-C** images from a 56-year-old normal subject without retina diseases. The en face OCT located superior temporal side of the macular. A large number of MLCs distributed evenly in this region. **D-F** images from a 69-year-old NPDR patient with DME. The en face OCT located superior temporal side of the macular. Compared with normal eyes C, more MLCs were found in this NPDR eye. **G-I** images from a 69-year-old PDR patient with DME. The en face OCT was located inferior nasal side of the macular. Numerous MLCs gathered around retinal neovascularization (yellow triangles on **G** & **H**). Some cells overlapped with retinal vessels (red ellipses on **G** & **I**). **J-L** images from a 70-year-old PDR patient without DME. The en face OCT was located superior temporal side of the macular. Retinal neovascularization was shown with yellow triangles on **J** & **K**. Some cells also overlapped with retinal vessels (red ellipses on **J** & **L**). MLC density decreased in superior retina and increased significantly in regions close to the macula

In addition, we investigated morphological and distribution characteristics of MLCs. MLCs appeared as small protrusions at the VRI on B scans (Fig. 4A, E, F, G, K&L). MLCs Cells in PDR and some NPDR eyes with DME gathered in clusters and moved closer to the fovea (Fig. 3C, G, D&H; Fig. 4A-D&G-J), which was quite different from normal eyes and NPDR eyes without DME (Fig. 3A, E, B&F). In some affected eyes, MLCs distributed along with the retinal vasculature (red ellipses in Fig. 4B, Fig. 5G, I, J&L). We also found that the distribution of MLCs in the retina was not limited to the macular region. We took en face OCT images of the middle periphery of the retina by guiding eye position from some subjects. We found that more MLCs distributed evenly in the peripheral retina in normal individuals and NPDR patients (Fig. 5A-C&D-F). In PDR eyes, MLCs gathered around the neovascularization and some MLCs distributed along with peri-vascular region (Fig. 5G-I&J-L).

## Discussion

In the current study, we used en face OCT image to investigate the density changes of MLCs located at the VRI in NPDR and PDR eyes. We found that PDR and DME were two factors that increased MLC density in the macular area. The study also demonstrated an association between VD and MLC density. To our best knowledge, this is the first large cohort study suggesting that MLC density increased in PDR and DME eyes.

The results that MLC density increased in DR especially PDR eyes is consistent with the previous study with a small sample size [25]. The uneven distribution of MLCs in fovea and periphery area is also in agreement with previous study [17, 18]. Pathology study on human retina found that cells located at the VRI were labelled by microglia markers in DR eyes. And the microglia were markedly increased in number and clustered around retina vessels at different stages of DR eyes and retinas with cystoid macular edema [10]. Distribution characteristic of MLCs in our study is similar with human histological investigations of macrophages. Hammer et.al [18] found that MLCs had close morphological and functional correspondence to true retinal microglia embedded within the neuropil. Other previous studies on animal models provided numerous evidence that microglial cell activation and pro-inflammatory molecules played a role in the development and progression of DR [5, 21, 27]. Although it is hard to completely illustrated the origin and the function of these MLCs in the current clinical study, our findings followed the previous experimental conclusions that the inflammatory response and immune cells takes part in the pathological process of DR.

Although more MLCs were found in the en face OCT centered on fovea in most DR eyes, we found that PDR and DME are two factors that significantly increase MLC density. PDR pathogenesis involved in not only severe ischemia burden but the injury of neurons and glial cells, dysfunction of endothelial progenitor cells and activation of inflammatory cells [26, 28]. The vitreous fluid in PDR eyes was rich of extracellular matrix, soluble proteins, and macromolecules, reproducing the soluble microenvironment of growth factors, cytokines, and metabolites [29]. Some inflammatory cells recruited to the retina produced angiogenic cytokines and effectors. These abnormal responses leaded to hemorrhage, hypoxia, fibrosis, further tissue damage, and retinal neovascularization [30]. Recruited and residential macrophages have been described to contribute to lymphatic vascular formation in inflammatory corneal neovascularization mouse models [31]. Recently, some researchers found microglia was the major cell population in surgically harvested vitreous fibrovascular membranes in PDR eyes [32]. Previous experimental and clinical studies demonstrated that inflammation and macrophages played a role in retinal neovascularization and inflammatory response, which is consistent with our results that MLC density increased in PDR eyes. However, the current study didn’t figure out the causal relationship between increased MLC density and neovascular occurrence. More research is needed to explain the mechanism behind phenomenon.

DME, a critical pathological hallmark, often accompanies all stages of DR and results in the vision loss and economic burden in DR patients. Macular edema results from an imbalance between fluid entry and drainage. The increase of vascular permeability, vessels abnormalization and neovascularization lead to more fluid entry the retina [33]. Numerous cytokines, chemokines and permeating factors have been repeatedly found elevated in ocular fluids of eyes with DME [34–37]. In addition, inflammatory pathways clearly involving in DME was evidenced by the clinical efficacy of intravitreal corticosteroid agents in DME [38]. Studies in diabetic rat retinas and human retinal endothelial cells both have demonstrated that monocyte macrophages which were recruited and infiltrated in retinal tissues leaded to dysfunction of the BRB [39]. On the other hand, researchers also suggested that microglia migrate to the outer retina causing alteration of RPE functions and caused subclinical retinal chronic inflammation which created environmental conditions for macular edema development [40]. In the current study, our finding, that MLC density increased a lot in eyes with DME, is consistent with previous experimental results and provide clinical evidence that macrophage/microglia may participate in DME pathological process. Our previous study in retinal vein occlusion also showed that MLC density correlated positively with retina thickness in chronic patients [19]. Another interesting result is that the positive correlation between MLC density and retinal thickness was established only in NPDR group. The presence or absence of DME had little impact on MLC density in the PDR eyes. We estimated that compared with those NPDR without DME, there are more severe inflammatory response, more MLCs recruitment and activation in NPDR eyes with DME. While in PDR eyes, immune cells have been activated and recruited and microenvironment is rich of growth factors, cytokines. In this situation, DME may have little influence on MLC density.

Another result in the current study is that MLC density correlates negatively with VD of DCP in NPDR eyes. It has been known that the DCP is more susceptible to ischemic damage and more vulnerable to injury because the deep layer of the retinal circulation sits next to high oxygen requirements of the outer plexiform layer [41, 42]. Large number of studies illustrated that VD of DCP significantly associated with DR progression [43, 44]. The negative correlation in our study may derive from the relation between VD of DCP and DR severity. However, previous study found that VD of SCP obtained from OCTA images can vary with scan pattern and scanned area. Also, there are artefacts that can influence the measurement of VD in OCTA images [45, 46]. Therefore, our findings in relationship between MLC density and VD need to be treated with caution.

There are some limitations in our study. First, there are some potential factors that may influence MLCs distribution and MLC density including hypertension, dyslipidemia, diabetes type and hemoglobin A1c. We didn’t include these factors in this study due to insufficient data. Second, we didn’t find the influence of PRP on MLC density. And we didn’t include subjects receiving intravitreal injection of steroids or anti-VEGF therapy within 3 months. Considering the inconsistent time interval between observation and PRP treatment in the retrospective study, the current study cannot draw any definite conclusion about the relationship between MLCs and PRP treatment. Third, although we acquired some images of the peripheral retina, the images were insufficient for statistical analysis. And the morphology and distribution were described rather than analyzed statistically. We thought that MLCs were not only located in the macular area but widely distributed at the VRI. Observations on a larger scale using wide-field en face OCT may help have a much better global picture of the dynamics and distribution of MLCs. Besides, we included patients with DME and analyzed the VD in the current study. The existence of DME and exudation may influence the segmentation and measurement of VD and contribute to the inaccurate results. To eliminate the effect, we examined the OCTA images and adjusted the segmentation manually if it was needed. Lastly, we found the increased level of MLCs density in DR. However, the discovery is insufficient to establish a causative pathogenic link. Further experimental studies are needed to explore the mechanism, the origin and the function of the cells.

## Conclusions

PDR and DME are two factors associated with the significantly increasing number of MLCs at the VRI. The current study provides an clinical insight that MLCs participate in DR progression and DME. The investigation of MLCs may be helpful for understanding their pathophysiology and finding new therapeutic targets for DR. The observation of MLCs provides a non-invasive way to evaluate the inflammatory response in vivo. Considered as a biomarker, MLCs might be useful in estimating the prognosis of DR and guide treatment in the clinic.

## Supplementary Information

**Additional file 1.**

## Data Availability

The datasets used during the current study are available from the corresponding author on reasonable request.

## Abbreviations

MLC: Macrophage-like cell
OCT: Optical coherence tomography
VRI: Vitreoretinal interface
DR: Diabetic retinopathy
GEE: Generalized estimating eqs.
SS-OCTA: Swept-source optical coherence tomography angiography
NPDR: Non-proliferative diabetic retinopathy
PDR: Proliferative diabetic retinopathy
VD: Vessel density
DME: Diabetic macular edema
DCP: Deep capillary plexus
SCP: Superficial capillary plexus
BRB: Blood–retinal barrier
PRP: Panretinal photocoagulation
