# Supplementary material for: Proliferative diabetic retinopathy and diabetic macular edema are two factors that increase macrophage-like cell density characterized by en face optical coherence tomography
Source: BMC Ophthalmol. 2023 Feb 1;23:46. doi: 10.1186/s12886-023-02794-8 (PMC9890872; doi:10.1186/s12886-023-02794-8)
Supplement: Supplementary file 1 — Additional file 1. [file 12886_2023_2794_MOESM1_ESM.docx]

We first derived the en face OCTA images of the MLC layer from the software and

then imported the images into ImageJ software for cropping and preprocessing. Since the en face macrophage layer was obtained through screenshots, we first clipped outside of the MLC images. Second, the square (radius = 5), horizontal line (radius = 10), vertical line (radius = 10), line 45 degrees (radius = 10), and line 135 degrees (radius = 10) in the white top hat algorithm were successively applied to enhance MLC imaging and to remove some artifacts. Larger artifacts were further removed through the subtract background (rolling = 10). Finally, the image in the previous step was copied and Gaussian blur was applied (sigma = 10). After subtracting the Gaussian blur from the image in the previous step, we obtained the preprocessed macrophage images. To extract MLCs from the images, several images with clear macrophage structure were selected and trainable WeKa segmentation was used to mark MLCs and the background. After that, an ideal training model was obtained, which was then used to extract MLCs from the preprocessed image. The resulting image was first converted to an 8-bit image and then binarized. The analyze particles tool was used to analyze the number, area, and percentage of MLCs.

FIJI Macro program:

rename("raw_image");

run("8-bit");

run("Morphological Filters", "operation=[White Top Hat] element=Square radius=5");

run("Morphological Filters", "operation=[White Top Hat] element=[Horizontal Line] radius=10");

run("Morphological Filters", "operation=[White Top Hat] element=[Vertical Line] radius=10");

run("Morphological Filters", "operation=[White Top Hat] element=[Line 45 degrees] radius=10");

run("Morphological Filters", "operation=[White Top Hat] element=[Line 135 degrees] radius=10");

rename("MLCs");

run("Subtract Background...", "rolling=10");

run("Duplicate...", " ");

run("Gaussian Blur...", "sigma=10");

imageCalculator("Subtract create", "MLCs","MLCs-1");

selectWindow("MLCs");

close();

selectWindow("MLCs-1");

close();

selectWindow("raw_image-White-White-White-White Top Hat");

close();

selectWindow("raw_image-White-White-White Top Hat");

close();

selectWindow("raw_image-White-White Top Hat");

close();

selectWindow("raw_image-White Top Hat");

close();

selectWindow("Result of MLCs");
